# Anti-glycaemic effect of the Chinese modified DASH diet combined with 23% low-sodium salt in patients with hypertension and type 2 diabetes: a clinical trial

**DOI:** 10.1186/s13098-025-01618-7

**Published:** 2025-02-12

**Authors:** Jiaxian An, Guowei Liu, Wenjun Luo, Xiaomeng Zhou, Ying Mei, Ziyan Zhang, Li Zhao, Yao Huang, Lihong Mu

**Affiliations:** 1 Department of Epidemiology, School of Public Health, Research Center for Medicine and Social Development, Chongqing Medical University, Chongqing, China; 2 Center for Disease Control and Prevention, Nanan District, Chongqing, China; 3 Center for Disease Control and Prevention, Fengjie County, Chongqing, China; 4 Health management center of the Second Affiliated Hospital of Chongqing Medical University, Chongqing, China

**Keywords:** Type 2 diabetes, Blood glucose, Hypertension, Low-sodium salt, Dietary approaches to stop hypertension diet, Glycaemic control rate

## Abstract

**Background:**

Although many previous trials have formalized the blood glucose-lowering effect of the DASH diet, relevant reports in China remain limited. This study aimed to explore the anti-hyperglycaemic effect of the Chinese Modified Dietary Approaches to Stop Hypertension diet combined with 23% low-sodium salt and meal packs in patients with hypertension and type 2 diabetes.

**Methods:**

We conducted a randomized controlled single-blinded trial with a semi-open design; 100 participants were randomly assigned to Group A (control), Group B (23% low-sodium salt), and Group C (meal packs) for 8 weeks of dietary intervention. All participants were followed up weekly to collect glycaemia data (standardized meal tolerance test), salt use, and adverse events.

**Results:**

Generalized estimating equation analysis indicated that fasting blood glucose decreased in all three groups following the intervention when compared to baseline. Group A decreased by 0.72 mmol/L (*P* = 0.008), while Groups B and C decreased by 2.02 mmol/L and 2.06 mmol/L, respectively (both *P* < 0.001). Although the latter two groups experienced greater reductions than Group A, the differences among the groups were not statistically significant (*P* = 0.450). For postprandial blood glucose, Group C showed the most pronounced decrease. The three groups recorded reductions of 2.43 mmol/L, 2.52 mmol/L, and 4.29 mmol/L, respectively (all P < 0.001), with again no significant difference observed between the groups (*P* = 0.088). The most notable enhancement in postprandial glucose was observed in Group C, which demonstrated a 51.5% improvement in its control rate. However, there was no statistically significant difference between the groups. No serious adverse events occurred during the trial.

**Conclusion:**

The CM-DASH diet combined with 23% low-sodium salt and meal packs demonstrates potentially beneficial effects on glycemic control in patients with hypertension and type 2 diabetes. This intervention reduces salt intake and fosters the development of healthy eating habits, thereby contributing to the improvement of patients' blood glucose. However, larger studies are necessary to confirm these findings.

*Trial registration* ChiCTR2000029017. Registered January 11, 2020-Prospective registration, http://www.chictr.org.cn/

## Background

Hypertension and diabetes are globally prevalent chronic non-communicable diseases [1], which not only share common pathogenic factors, but also influence each other. In China, nearly 60% of individuals with diabetes also experience elevated blood pressure [2], and about 35–75% of diabetic complications are linked to hypertension [3]. The development of hypertension and diabetes is closely related to poor dietary habits, such as low potassium and high sodium diets [4, 5]. Therefore, lifestyle modifications, particularly dietary interventions, are essential for effective disease management.

The low-sodium, high-potassium diet represents a well-balanced dietary pattern, with the Dietary Approaches to Stop Hypertension (DASH) diet in the United States being a prime example.The DASH diet is effective in lowering blood pressure among patients with hypertension by promoting foods rich in potassium, low in sodium, and high in dietary fiber [6, 7]. Although initially designed to address hypertension, the benefits of the DASH diet have extended to other health problems such as metabolic syndrome and diabetes [8–11]. A systematic review examining multiple prospective cohort studies conducted in Europe revealed that each unit increase in the DASH dietary index was linked to an 8–18% reduction in the risk of developing type 2 diabetes [12]. Furthermore, its relationship with the risk of developing type 2 diabetes and the cardiometabolic risk to patients has been demonstrated [11, 13]. A 6-month study demonstrated that hypertensive patients adhering to the DASH diet showed a more pronounced decrease in fasting glucose compared to the control group (− 0.53 mmol/L vs − 0.14 mmol/L) [14]. This finding was corroborated by an additional 8-week randomized crossover clinical trial, which revealed a notably greater decrease in fasting blood glucose among participants following the DASH diet than those in the control group (− 29.4 mg/dl vs − 12.8 mg/dl) [15].

Although many previous trials have formalized the blood glucose-lowering effect of the DASH diet, relevant reports in China remain limited. Chinese residents typically experience excessive sodium intake, largely due to their preference for salty foods. Salt consumption often surpasses the daily limits recommended by the World Health Organization [16]. Based on the characteristics of Chinese adults, the Chinese Nutrition Society recommends a maximum salt intake of 6 g/day. However, findings from the NTERMAP study indicate that the average salt consumption among the Chinese population is significantly higher, at 13.3 g/day [17]. This dietary pattern pose challenges to the promotion of the DASH dietary approach within China. Consequently, there is a need for localized adaptations of the DASH diet to better align with Chinese culinary preferences and cultural practices.

In this study, we utilized low sodium salt (containing 23% NaCl) and meal packs to investigate their effects on blood glucose levels and glycemic control in conjunction with the Chinese Modified DASH (CM-DASH) diet. The objective was to evaluate the efficacy of this dietary pattern on evidence-based glycemic control in hypertensive and type 2 diabetic patients taking regular medication.

## Materials and methods

### Sample size

Based on the pre-test data estimation [18–20], 30–40 participants were included in each group, totalling approximately 90–120 cases.

### Participants

**Fig. 1:**
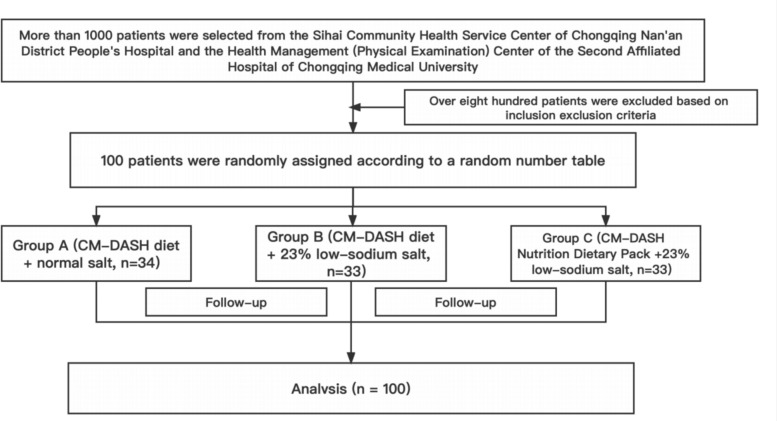
Flowchart of the study

This trial was conducted in Chongqing, China, between July 2021 and December 2021; 100 participants who were eligible for the test were selected from the Sihai Community Health Service Center of Chongqing Nanan District People's Hospital and the Health Management (Physical Examination) Center of the Second Affiliated Hospital of Chongqing Medical University. The flow diagram depicting the enrolment of the participants in the study is shown in Fig. 1.

#### Inclusion criteria

The inclusion criteria were: (1) age between 50 and 75 years; (2) residence near the hospital without any plan to change residence or leave the city during the trial; (3) patients with type 2 diabetes and hypertension comorbidity, currently on consistent antihypertensive and anti-glycaemic medications, adhering to the criteria outlined in the "Chinese Guidelines for the Prevention and Control of Hypertension, Revised Edition, 2018" [21] and the diagnostic criteria for diabetes as per the "Chinese Guidelines for Prevention and Control of Type 2 Diabetes, 2017 Edition" [22]; (4) commitment to strictly consuming the two daily meals provided during the trial; (5) signing of informed consent by the participants and their cohabitating family members.

#### Exclusion criteria

We excluded the following from this study: (1) individuals with malignant tumours, recent acute-phase heart attack, stroke within the past 3 months, or other serious illnesses with a life expectancy of less than 1 year; (2) individuals with hypercortisolism or aldosteronism; (3) those experiencing acute-phase illnesses such as upper respiratory tract infections, fever, severe diarrhoea; (4) those with hearing impairments, dementia, or other disabilities that hinder normal communication, as well as severe depression or other mental disorders; (5) individuals with mobility issues preventing timely follow-up, or failure to complete follow-up after enrolment; (6) patients with chronic renal failure of stage 3 or higher, or undergoing renal replacement therapy; (7) individuals with abnormal liver function, evidenced by alanine aminotransferase or aspartate aminotransferase levels more than twice the upper limit of normal, or total bilirubin levels exceeding the normal upper limit; (8) individuals with abnormal potassium levels, below 3.5 mmol/L or above 5.5 mmol/L, or those taking potassium-sparing diuretics; (9) pregnant individuals or those likely to become pregnant, and others unsuitable for the trial product; (10) individuals consuming low-sodium salt or participating in other clinical studies deemed inappropriate for trial inclusion by the investigator; and (11) individuals considered unsuitable for enrolment with the trial product by the investigator.

### Study design

This study used a randomized, controlled, single-blind trial with a semi-open design. A total of 100 patients with hypertension and type 2 diabetes were recruited and enrolled through community hospitals. The participants were allocated into three groups: Group A, the control group (CM-DASH diet + common salt, n = 34); Group B, the 23% salt group (CM-DASH diet + 23% sodium-restricted formula salt, n = 33); and Group C, the meal packs group (CM-DASH technology package + 23% sodium-restricted formula salt, n = 33).

Salt consumption was maintained at 5 g/day per capita in all groups, aligning with World Health Organization-recommended salt standards. After completing the baseline survey and physical examination, an 8-week dietary intervention was initiated. This intervention comprised the dietary guidance phase during weeks 1 and 2 (distribution of salt + CM-DASH dietary recipes), centralized meal feeding phase during weeks 3 and 4 (meals provided in the hospital cafeteria), and home health care phase during weeks 5–8 (distribution of salt + CM-DASH dietary recipes). Weekly follow-up visits were conducted to gather data on blood glucose levels, salt consumption, and adverse events, as well as to reinforce adherence to recommended salt intake and dietary guidelines. Questionnaires and physical examinations were repeated at week 4 and at the conclusion of the intervention.

#### CM-DASH recipes

According to the recommendations of the Chinese Guidelines for the Prevention and Control of Type 2 Diabetes (2018 Edition) and the Chinese Guidelines for the Prevention and Control of Hypertension (2017 Revision), respectively, patients with diabetes should adopt a diversified dietary pattern that is "cereal-based, with a high intake of dietary fibre, and low in salt, sugar, and fat," whereas patients with hypertension should consume mainly fruits, vegetables, low-fat dairy products, whole grains rich in dietary fibre, and vegetable-derived protein, and reduce the intake of saturated fat and cholesterol. The researchers formulated a collection of recipes tailored for Chinese patients suffering from hypertension and type 2 diabetes, drawing upon the dietary habits prevalent within the Chinese population. This approach aligns with the strategies outlined in that paper [20]. All participants cooked food at home (during the first, second, and fifth to eighth weeks) using the recommended recipes (Table 1).

**Table 1:** Nutrient composition of CM-DASH (Chinese Modified Dietary Approaches to Stop Hypertension) diet

Food		Net mass (g)	Energy (kcal)	Protein (g)	Fat (g)	Carbohydrates (g)	Dietary fiber(g)	Sodium (mg)	Potassium (mg)	Magnesium (mg)	Calcium (mg)
Breakfast	Whole-wheat bread	100.00	163.00	10.50	0	52.30	4.20	265.00	250.00	77.00	163.00
	Low-fat, high-calcium milk	250.00	110.00	7.50	3.25	12.25	0	180.00	272.50	27.50	312.50
	Egg	50.00	71.50	6.05	5.25	0.05	0	65.00	65.00	0	17.50
Lunch	Mixed grain rice	60.00	69.00	1.93	0.43	18.24	0.28	0.53	34.61	11.42	3.91
	Meat (chicken, fish)	60.00	59.20	12.09	1.11	0.18	0	38.04	213.90	17.70	0.60
	Vegetables (dark green vegetables)	250.00	46.25	3.19	0.56	10.19	4.13	38.75	463.13	43.75	60.00
Dinner	Mixed grain rice	60.00	69.00	1.93	0.43	18.24	0.28	0.53	34.61	11.42	3.91
	Meat (chicken, fish)	60.00	59.20	12.09	1.11	0.18	0	38.04	213.90	17.70	0.60
	Vegetables (dark green vegetables)	250.00	46.25	3.19	0.56	10.19	4.13	38.75	463.13	43.75	60.00
Others	Nut (walnut)	20.00	129.20	2.98	11.76	3.82	1.90	1.28	77.00	26.20	11.20
	Fruit	150.00	79.50	0.60	0.30	20.55	2.55	1.95	124.50	6.00	6.00
	Cooking oil	20.00	179.80	0	19.98	0	0	0.70	0.20	0.40	2.40
	Salt	5.00	48.77	0.22	0	0.46	0	1025.30	807.75	2.00	22.00
Total		1335.00	1130.67	62.27	44.74	146.65	17.46	1693.87	3020.23	284.84	663.62

#### Meal packs

The meal packs (CM-DASH Nutritional Meal Packs, produced by Chongqing Shanshun Biotechnology Co., Ltd., adhering to Product Standard GB/T29603; Food Production License No. SC12450023228393) are designed for the dietary treatment of type 2 diabetes and hypertension. They are tailored to the dietary structure and habits of Chinese people, incorporating principles of dietary management for hypertension and diabetes. This includes the selection of traditional Chinese medicines used in treating these conditions, as well as modern nutritional medicines for hypertension and diabetes. It uses mixed-grain and homogenized meals as the core of the nutritional intervention treatment and was provided as an intervention for Group C. It included the following items: CM-DASH Homogenized Meal Solid Drink (homogenized meal for breakfast), CM-DASH Solid Drink (Normal Companion), and CM-DASH Eight Treasures Rice (Rice for Mid-Dinner). The ingredients of each item are shown in Table 2.

**Table 2:** The meal packs (CM-DASH Nutrition Dietary Packs) composition

Nutrient content	CM-DASH Homogenized Meal Solid Drink (Homogenized Meal for Breakfast)	CM-DASH Solid Drink (Normal Companion)	CM-DASH Eight Treasures Rice (Rice for Mid-Dinner)
Weight (g)	39	60	200
Energy (kcal)	119.74	171.36	722.26
Nutrient content	CM-DASH Homogenized Meal Solid Drink (Homogenized Meal for Breakfast)	CM-DASH Solid Drink (Normal Companion)	CM-DASH Eight Treasures Rice (Rice for Mid-Dinner)
Protein (g)	8.4	8.8	38.4
Fat (g)	1.5	2.7	14.4
Carbohydrates (g)	17.7	27.5	118.8
Fiber (g)	—	—	14.97
Sodium (mg)	90	26	10
Potassium (mg)	—	—	—
Magnesium (mg)	60	—	—
Calcium (mg)	120	—	—

#### Salt use

The test salt was used throughout the trial as a substitute for the participants' household salt. Group A received common salt ("Jing Xin," manufactured by Chongqing Salt Industry Group Co., Ltd. Name: purified salt; product standard: NY/T1040; sodium chloride content: > 99%). Groups B and C received 23% low-sodium salt ("Shan Yi Kang" manufactured by Chongqing Shanshun Biotechnology Co., Ltd. Name: solid compound condiment, standard of execution: Q/SWS 0025S, food production license number: SC10650012000709; main ingredients include potassium chloride [56%], sodium chloride [23%], and protein [3.0%]). We provided participants with dosing spoons to help them manage their salt intake at home, ensuring it did not exceed 5 g per person per day. Additionally, an electronic scale with a precision of 0.1 g was utilized to monitor weekly salt consumption, from which the daily salt intake for each family member was estimated.

### Blood glucose measurement

Participants attended follow-up appointments in the hospital once a week. Glycaemia (standardized meal tolerance test) was measured by professionally trained researchers using a medical blood glucose meter (Dahl C type, GM505RAB). The researchers made the measurements strictly following the clinical application guideline for blood glucose monitoring in China (2021 edition). All participants were asked to complete a fasting blood glucose measurement at 8:00 a.m., and postprandial blood glucose was measured 2 h (counted from the first bite of breakfast eaten) after breakfast was distributed on site.

The glycaemic control rate was defined as a fasting blood glucose level of < 7.0 mmol/L and a postprandial blood glucose level of < 10.0 mmol/L.

### Statistical analysis

The Kolmogorov–Smirnov test was used to test the normality of the data, and the Levene test was used to test the chi-square of the groups. Normally distributed continuous variables were expressed as the mean ± standard deviation using the independent samples t-test; skewed continuous variables were expressed as median and interquartile range using the Kruskal–Wallis H test. Categorical variables were expressed as counts and percentages using Pearson's chi-square test or continuous corrected chi-square test. The continuously measured blood glucose value were analysed using generalized estimating equations. All statistical analyses were performed using SPSS 27.0 (IBM, Armonk, NY, USA) statistical software, and the tests were two-sided, with differences considered statistically significant at P < 0.05.

## Results

### Demographic characteristics

In total, 100 participants were included in this study. No significant difference was observed in the demographic characteristics except the age of the participants (Table 3).

**Table 3:** Baseline characteristics of participants

Characteristics	Group A(control group, n = 34)	Group B (23% low-sodium salt group, n = 33	Group C (meal packs group, n = 33)	*P*
Gender (Male, %)	16 (47.1)	17 (51.5)	16 (48.5)	0.933^a^
Age (y)	68.12 ± 3.95	63.39 ± 6.83	67.21 ± 5.19	0.001^b^*
BMI(kg/m2)	24.96 ± 2.12	25.46 ± 8.66	24.50 (22.50, 26.95)	0.580^c^
Waist circumference (cm)	84.41 ± 6.65	86.67 ± 8.06	86.36 ± 11.12	0.523^b^
Hip circumference (cm)	92.03 ± 5.56	94.00 (92.00, 98.50)	95.06 ± 8.56	0.124^c^
Waist-to-hip ratio	0.92 ± 0.05	0.90 ± 0.06	0.91 ± 0.06	0.605^b^
fasting blood glucose (mmol/L)	8.20 (5.90, 10.00)	8.30 (7.35, 10.30)	8.20 (7.80, 9.10)	0.386^c^
postprandial blood glucose (mmol/L)	11.85 (8.13, 13.23)	11.00 (8.30, 12.55)	11.00 (8.40, 14.00)	0.839^c^
Fasting blood glucose compliance rate (n,%)	14 (41.2)	6 (18.2)	3 (9.1)	0.006^a^
Postprandial blood glucose compliance rate (n,%)	15 (44.1)	12 (36.4)	14 (42.4)	0.540^a^
Using statins (n,%)	14 (41.2)	10 (30.3)	17 (51.5)	0.215^a^
Using aspirin (n,%)	8 (23.5)	6 (18.2)	8 (24.2)	0.809^a^
Family history of diabetes (n,%)	12 (35.3)	13 (40.6)	15 (48.4)	0.561^a^

^a^R*C Chi-square test for contingency tables

^b^one-way ANOVA

^c^Kruskal-Wallis H test for comparison of multiple samples with completely random design

^*^Group A VS. Group B: *P* = 0.001, Group B VS. Group C: *P* = 0.005

### Fasting glucose changes

Compared with baseline, all groups showed a downward trend in fasting glucose, with a mean decrease of 2.02 mmol/L (95% confidence interval CI − 3.01–− 1.04, *P* = 0.000) in Group B and 2.06 mmol/L (95% CI − 2.75–− 1.37, *P* = 0.000).

In Group C. The mean decrease in Group A was 0.72 mmol/L (95% CI − 1.25–− 0.19, *P* = 0.008), which was less than that in Groups B and C; no significant difference was observed in the comparison between the participant groups (Table 4).

**Table 4:** Changes in fasting plasma glucose from baseline during intervention

	Group A(control group, n = 34)			Group B(23% low-sodium salt group, n = 33			Group C(meal packs group, n = 33)			*P* **
	Blood glucose [M (P25, P75)]	Change(MD, 95%CI)	P*	Blood glucose [M (P25, P75)]	Change(MD, 95%CI)	*P* *	Blood glucose [M (P25, P75)]	Change(MD, 95%CI)	*P* *	
Baseline	8.20 (5.90, 10.00)	–	–	8.30 (7.35, 10.30)	–	–	8.20 (7.80, 9.10)	–	–	0.386
1w	7.80 (6.30, 8.85)	− 0.33 (− 0.83, 0.18)	0.204	6.50 (5.95, 8.55)	− 1.66 (− 2.52, − 0.80)	0.000	7.30 (6.45, 8.10)	− 1.37(− 1.91, − 0.82)	0.000	0.371
2w	7.45 (6.18, 8.88)	− 0.53 (− 1.05, − -0.01)	0.047	6.80 (6.25, 8.70)	− 1.60 (− 2.44, − 0.77)	0.000	7.10 (6.00, 7.80)	− 1.79(− 2.32, − 1.25)	0.000	0.516
3w	7.45 (5.85, 8.63)	− 0.76 (− 1.27, − 0.24)	0.004	6.30 (6.05, 7.55)	− 2.16 (− 3.02, − 1.29)	0.000	6.90 (6.20, 8.25)	− 1.62(− 2.15, − 1.10)	0.000	0.310
4w	7.45 (6.08, 9.18)	− 0.50 (− 0.98, − 0.03)	0.037	6.90 (5.80, 8.15)	− 1.86 (− 2.76, − 0.96)	0.000	7.00 (6.30, 8.35)	− 1.44(− 2.14, − 0.74)	0.000	0.497
5w	7.45 (6.15, 8.00)	− 0.74 (− 1.33, − 0.15)	0.014	6.40 (5.80, 7.95)	− 2.28 (− 3.23, − 1.34)	0.000	7.10 (6.05, 8.30)	− 1.74(− 2.45, − 1.02)	0.000	0.220
6w	7.10 (6.05, 8.73)	− 0.71 (− 1.23, − 0.18)	0.008	6.40 (5.95, 7.60)	− 2.09 (− 2.96, − 1.21)	0.000	6.80 (5.85, 7.90)	− 2.07(− 2.74, − 1.40)	0.000	0.350
7w	7.40 (5.98, 8.60)	− 0.85 (− 1.38, − 0.33)	0.001	6.50 (5.85, 7.45)	− 2.24 (− 3.22, − 1.26)	0.000	7.20 (6.25, 8.00)	− 1.81(− 2.49, − 1.13)	0.000	0.250
8w	7.35 (6.08, 8.28)	− 0.72 (− 1.25, − 0.19)	0.008	6.70 (5.80, 8.15)	− 2.02 (− 3.01, − 1.04)	0.000	6.80 (5.85, 7.85)	− 2.06(− 2.75, − 1.37)	0.000	0.450

^*^The baseline differences in age were adjusted using generalized estimation equatio

^**^Comparison between groups

### Postprandial blood glucose changes

At the conclusion of the intervention, postprandial blood glucose levels changed significantly in all groups compared with baseline. Within-group comparisons revealed a mean reduction in postprandial glucose of 2.43 mmol/L (95% CI − 3.43–1.43, *P* = 0.000) for group A, and a mean reduction of 2.52 mmol/L(95% CI − 3.94–1.10, *P* = 0.000) for group B. The most pronounced improvement in mean postprandial glucose was observed in group C, with a decrease of 4.29 mmol/L (95% CI − 5.67–2.90, *P* = 0.000). Notably, there were no statistically significant differences among the three groups (Table 5).

**Table 5:** Changes in 2 h postprandial blood glucose from baseline during intervention

	Group A(control group,n = 34)			Group B(23% low-sodium salt group,n = 33			Group C(meal packs group,n = 33)			*P* **
	Blood glucose [M (P25, P75)]	Change(MD, 95%CI)	*P* value*	Blood glucose [M (P25, P75)]	Change(MD, 95%CI)	*P* value*	Blood glucose [M (P25, P75)]	Change(MD, 95%CI)	*P* value*	
Baseline	11.85 (8.13, 13.23)	–	–	11.00 (8.30, 12.55)	–	–	11.00 (8.40, 14.00)	–	–	0.839
1w	10.45 (7.68, 13.13)	− 0.84 (− 1.43, − 0.24)	0.006	9.70 (7.85, 11.75)	− 0.42 (− 2.06, 0.25)	0.615	8.10 (6.80, 8.90)	− 3.45 (− 4.72, − 2.19)	0.000	0.012^a^
2w	10.35 (7.98, 12.68)	− 0.92 (− 1.73, − 0.12)	0.025	8.20 (6.55, 10.60)	− 2.27 (− 3.51, − 1.04)	0.000	7.90 (6.55, 9.25)	− 3.48 (− 4.71, − 2.24)	0.000	0.012^b^
3w	9.10 (7.38, 11.15)	− 1.77 (− 2.63, − 0.92)	0.000	9.50 (8.00, 11.35)	− 1.36 (− 2.53, − 0.18)	0.024	8.00 (7.05, 9.45)	− 3.25 (− 4.53, − 1.96)	0.000	0.102
4w	9.35 (6.95, 11.80)	− 1.74 (− 2.68, − 0.80)	0.000	8.30 (6.90, 10.45)	− 1.67 (− 3.20, − 0.15)	0.031	7.90 (6.70, 9.90)	− 3.25 (− 4.58, − 1.91)	0.000	0.291
5w	9.20 (7.85, 11.85)	− 1.63 (− 2.62, − 0.63)	0.001	8.30 (6.90, 10.60)	− 1.76 (− 3.27, − 0.25)	0.023	7.80 (6.35, 9.55)	− 3.49 (− 5.02, − 1.97)	0.000	0.067
6w	9.40 (7.48, 10.95)	− 1.70 (− 2.61, − 0.79)	0.000	9.20 (7.50, 10.30)	− 1.60 (− 2.91, − 0.30)	0.016	7.00 (6.45, 8.25)	− 4.14 (− 5.62, − 2.67)	0.000	0.001^c^
7w	8.65 (7.13, 11.08)	− 2.09 (− 3.01, − 1.16)	0.000	8.50 (6.45, 10.10)	− 2.47 (− 3.80, − 1.14)	0.000	7.50 (6.65, 8.90)	− 3.81 (− 5.33, − 2.29)	0.000	0.140
8w	8.25 (7.05, 10.25)	− 2.43 (− 3.43, − 1.43)	0.000	7.50 (6.70, 9.75)	− 2.52 (− 3.94, − 1.10)	0.000	7.30 (6.60, 8.20)	− 4.29 (− 5.67, − 2.90)	0.000	0.088

^*^The baseline differences in age were adjusted using generalized estimation equations

^**^Comparison between group

^a^Group C VS Group B:*P* = 0.032

Group C VS Group A:*P* = 0.028

^b^Group C VS Group A:P = 0.017

^c^Group C VS Group B:*P* = 0.004

Group C VS Group A:*P* = 0.002

### Change in glycaemic control rate

After eight weeks of intervention, glycemic control improved by 42.4% and 39.4% in group B, and by 42.4% and 51.5% in group C, compared to baseline. No statistically differences were observed between the groups (Table 6).

**Table 6:** Changes in glycemic control rate from baseline during intervention

	Group A(control group,n = 34)				Group B(23% low-sodium salt group, n = 33				Group C (meal packs group, n = 33)				Fasting plasma glucose	2 h postprandial blood glucose
	Fasting plasma glucose		2 h postprandial blood glucose		Fasting plasma glucose		2 h postprandial blood glucose		Fasting plasma glucose		2 h postprandial blood glucose			
	Number	Ratio (%)	Number	Ratio (%)	Number	Ratio (%)	Number	Ratio (%)	Number	Ratio (%)	Number	Ratio (%)	P*	P**
Baseline	14	41.2	15	44.1	6	18.2	12	36.4	3	9.1	14	42.4	0.006^a^	0.796
1w	13	38.2	16	47.1	21	63.6	18	54.5	13	39.4	27	81.8	0.065	0.009^c^
2w	11	32.4	16	47.1	18	54.5	22	66.7	15	45.5	27	81.8	0.184	0.011^d^
3w	14	41.2	20	58.8	22	66.7	21	63.6	18	54.5	26	78.8	0.112	0.195
4w	14	41.2	19	55.9	17	51.5	23	69.7	16	48.5	27	81.8	0.618	0.071
5w	12	35.3	22	64.7	23	69.7	24	72.7	14	42.4	26	78.8	0.012^b^	0.436
6w	15	44.1	21	61.8	19	57.6	20	60.6	17	51.5	30	90.9	0.544	0.009^e^
7w	15	44.1	23	67.6	21	63.6	25	75.8	13	39.4	28	84.8	0.112	0.257
8w	14	41.2	25	75.5	20	60.6	25	75.8	17	51.5	31	93.9	0.282	0.067

^*^Comparison between Fasting plasma glucose

^**^Comparison between 2 h postprandial blood glucose

^a^Group A VS Group C:*P* = 0.003

^b^Group A VS Group B:*P* = 0.005

^c^Group A VS Group C:*P* = 0.003

^d^Group A VS Group C:*P* = 0.003

^e^Group A VS Group C:*P* = 0.005;Group B VS Group C:*P* = 0.004

### Adverse effects

No serious adverse reactions occurred during the intervention. During the follow-up, a total of eight participants (8.0%) (two [5.88%] in Group A, one [3.03%] in Group B, and five [15.15%] in Group C) exhibited mild symptoms, such as dizziness, fatigue, diarrhoea, and abdominal pain. All adverse events were short-term, and symptoms resolved spontaneously (Table 7).

**Table 7:** Adverse events

No	Group	Symptoms	Time (Week)	Measures	Transfer to
120206	A	Blurred eyes, Regurgitation	1	–	Recovery
121007	A	Diarrhea	3	–	Recovery
092807	B	Lack of power	2	Use of common salt	Recovery
092737	C	Indigestion	2	Take medication (Morpholine)	Recovery
092834	C	Hypoglycemia	2	Eating cookies	Recovery
092832	C	Hypoglycemia	2	Eating cookies	Recovery
092711	C	Hypoglycemia	2	Reduce glucose-lowering medication as prescribed by doctor	Recovery
092710	C	Diarrhea	3	Take medication (Norfloxacin)	Recovery

## Discussion

The objective of this study was to examine the antihyperglycemic effect of CM-DASH diet combined with 23% low sodium salt and meal packs in patients with hypertension and type 2 diabetes The prevalence of diabetes is higher in patients with hypertension than in the general population [2], and the degree of target organ damage is more severe in patients with hypertension and diabetes than in patients with hypertension alone [18]. Therefore, early implementation of dietary interventions in patients with hypertension and diabetes is essential to control blood pressure and glucose. However, the current approach to hypertension and diabetes control in China is mainly through pharmacological treatment, and the control rate of hypertension and diabetes is limited owing to the poor compliance of patients with medication and the lack of lifestyle interventions. Therefore, it is necessary to introduce effective dietary interventions to patients with hypertension and diabetes to reduce the associated risks. Different dietary patterns exert varying impacts on health. The Eastern dietary pattern, which emphasizes the consumption of vegetables and fruits while maintaining a low intake of saturated fats, along with the Mediterranean dietary pattern—characterized by a focus on olive oil, whole grains, vegetables, and moderate amounts of fish—has been associated with numerous health benefits. These include reductions in blood pressure, improvements in blood glucose levels, and decreased risk of chronic kidney disease [23–26]. In contrast, the Western dietary pattern, characterized by a high intake of red meat, processed meats, saturated fat and low dietary fiber, may elevate the risk of diabetes and gout [27, 28]. Although the Eastern and Mediterranean dietary patterns are globally recognized for their health benefits, their effects are also influenced by geography. People in different regions may exhibit varying preferences and acceptance of specific foods;thus, these considerations must be accounted for when promoting healthy dietary patterns to ensure that dietary recommendations are widely accepted and consistently implemented. This study adopted a Chinese-modified DASH dietary pattern tailored to the dietary culture and habits of the Chinese population. This modification not only preserves the advantages of the DASH diet in lowering blood pressure, improving glycemic control, and promoting cardiovascular health but also enhances the feasibility and acceptability of the dietary pattern by adjusting food types and proportions to better align with traditional Chinese eating habits.

This study demonstrated that, compared to the initial blood glucose values, participants in all three groups experienced significantly greater reductions in both fasting and postprandial blood glucose levels following the implementation of the CM-DASH dietary pattern. This finding underscores the effectiveness of the CM-DASH dietary approach in lowering blood glucose levels. Further analysis indicated that groups B and C exhibited a more pronounced reduction in blood glucose compared to group A, suggesting that the CM-DASH diet combined with 23% low-sodium salt may be more effective for reducing blood glucose levels than using regular table salt. In addition, group C exhibited the most significant reduction in postprandial glucose levels compared to baseline. This effect is likely attributable to the meal packs products, which contained a substantial amount of dietary fiber, no added sugars, and a more comprehensive nutritional profile. Dietary fibre can enhance insulin secretion or sensitivity, increase glucose uptake, inhibit lipid accumulation and hepatic gluconeogenesis, and improve oxidative stress levels to achieve the hypoglycaemic effect [29]. Therefore, we suggest that the addition of the meal packs to a low-sodium salt diet may be more beneficial for the control of postprandial glucose in patients. Postprandial glucose levels play a significant role in cardiovascular events and serve as an independent risk factor for such events in patients with type 2 diabetes [30]. Postprandial glucose also reflect the level of glucose tolerance in patients with diabetes, which is usually assessed using 2-h postprandial glucose [31], which is extremely important for both early diagnosis and control of diabetes, and changes in dietary habits can directly affect postprandial glucose levels [32]. However, due to the absence of statistically significant differences in the between-group comparisons, we are unable to determine which intervention was more effective. This limitation may be attributed to the small sample size.

A survey of hypertension and diabetes in residents aged 35–75 years in Chongqing, China, showed that the glycaemic control rate in patients with hypertension and diabetes was 16.17%, which remains low given the large number of patients [33]. At baseline in this trial, the fasting glucose control rate for all patients was 23.0%, which is generally consistent with the results of the above survey. At the end of the intervention, the fasting glucose control rate increased by 42.4% from baseline in both Groups B and C. The highest rates were reached in Group B at week 5 (69.7%) and Group C at week 3 (54.5%), with an increase of 51.5% and 45.5% from baseline, respectively. Further, the postprandial glucose control rate improved even more significantly during the intervention period, from 44.1%, 36.4%, and 42.4% at baseline to 75.5%, 75.8%, and 93.9% at the end of the intervention in the three groups, respectively, all reaching the highest value at 8 weeks. This result reinforces the use of the meal packs in combination with low-sodium salt as more effective intervention for controlling postprandial glucose in patients and that this effect may become more significant as the duration of the intervention increases. However, this study found no statistically differences among the various groups, indicating that further research is necessary to validate these findings.

Regarding adverse effects, no serious complications were reported in this study; mild symptoms such as dizziness, malaise, diarrhea, and abdominal pain were transient and resolved spontaneously. These results suggest that the CM-DASH diet combined with low-sodium salt and meal packs represents a safe intervention, which is crucial for ensuring long-term adherence to dietary modifications by patients.

Due to the semi-open experimental design of this trial, the results are inevitably affected by interference, contamination, and other factors. There were some fluctuations in the blood glucose of the patients during the trial; however, the results consistently indicated that the dietary intervention had some health benefits for patients with hypertension and type 2 diabetes. The persistent nature of chronic diseases presents a significant challenge to global healthcare systems. Patients often experience prolonged periods of poor health and require long-term medical care [34]. Therefore, large-scale dietary interventions and health education initiatives can be implemented within community-based populations to promote the use of low-sodium salt in combination with meal packs in specific populations. This approach aims to enhance knowledge regarding diabetes management among these populations, ultimately reducing the burden of disease.

There are several limitations to this study. Firstly, the small sample size prevents conclusions about which intervention is more effective. The smaller sample size may also affect the generalizability and statistical significance of the findings. Secondly, the research was based on a specific population in Chongqing, China, and may not be directly generalizable to populations in other regions. More studies are needed to validate the effects of this dietary interventions in different populations. Thirdly, the study's short duration may restrict the assessment of long-term effects and sustainability. Effective management of chronic diseases necessitates long-term follow-up; thus, short-term studies may not adequately capture enduring outcomes. Fourthly, while although this paper adjusts for age, it is still possible that it may have had an impact on the results,particularly considering the influence of age on metabolic health. Additionally, other lifestyle factors that might impact glycemic control—such as physical activity and sleep—were not addressed in this study. Future studies should incorporate these variables to provide a more comprehensive and accurate assessment.

## Conclusions

In summary, this study demonstrates that the CM-DASH diet combined with 23% low-sodium salt and meal packs has potentially beneficial effects on glycemic control in patients suffering from hypertension alongside type 2 diabetes. However, this study is merely a preliminary investigation; therefore, future research should expand the sample size for long-term follow-up in order to further elucidate the role of this dietary pattern in the long-term management of diabetes. Additionally, consideration of the cultural and dietary habits of diverse populations is essential for developing more specific and acceptable dietary interventions.

## Data Availability

No datasets were generated or analysed during the current study.
